# Tuning the Defects of Two-Dimensional Layered Carbon/TiO_2_ Superlattice Composite for a Fast Lithium-Ion Storage

**DOI:** 10.3390/ma15051625

**Published:** 2022-02-22

**Authors:** Bingheng Liu, Bo Gu, Jingxian Wang, Anchang Li, Ming Zhang, Zhongrong Shen

**Affiliations:** 1 College of Chemistry, Fuzhou University, Fuzhou 350108, China; binghengliu@fjirsm.ac.cn; 2 CAS Key Laboratory of Design and Assembly of Functional Nanostructures, and Fujian Key Laboratory of Nanomaterials, Fujian Institute of Research on the Structure of Matter, Chinese Academy of Sciences, Fuzhou 350002, China; xmgubo@fjirsm.ac.cn (B.G.); wangjingxian_19@163.com (J.W.); xmlianchang@fjirsm.ac.cn (A.L.); 3 Xiamen Key Laboratory of Rare Earth Photoelectric Functional Materials, Xiamen Institute of Rare Earth Materials, Haixi Institute, Chinese Academy of Sciences, Xiamen 361021, China

**Keywords:** nanosheets, carbon/TiO_2_, superlattice, defects, reduced titanium

## Abstract

Defect engineering is one of the effective ways to improve the electrochemical property of electrode materials for lithium-ion batteries (LIB). Herein, an organic functional molecule of p-phenylenediamine is embedded into two-dimensional (2D) layered TiO_2_ as the electrode for LIB. Then, the 2D carbon/TiO_2_ composites with the tuning defects are prepared by precise control of the polymerization and carbothermal atmospheres. Low valence titanium in metal oxide and nitrogen-doped carbon nanosheets can be obtained in the carbon/TiO_2_ composite under a carbonization treatment atmosphere of N_2_/H_2_ gas, which can not only increase the electronic conductivity of the material but also provide sufficient electrochemical active sites, thus producing an excellent rate capability and long-term cycle stability. The prepared composite can provide a high capacity of 396.0 mAh g^−1^ at a current density of 0.1 A g^−1^ with a high capacitive capacity ratio. Moreover, a high specific capacity of 80.0 mAh g^−1^ with retention rate of 85% remains after 10,000 cycles at 3.0 A g^−1^ as well as the Coulomb efficiency close to 100%. The good rate-capability and cycle-sustainability of the layered materials are ascribed to the increase of conductivity, the lithium-ion transport channel, and interfacial capacitance due to the multi-defect sites in the layered composite.

## 1. Introduction

The development of the next-generation electrode materials for high-power density LIB is one of the main domains that we urgently need to focus on. Two-dimensional metal oxides are considered to be one kind of potential electrode material for high-performance LIB due to their short active paths and highly exposed active sites on the surface. However, the low electronic conductivity seriously restricts their electrochemical properties [1,2,3,4,5]. Therefore, the combination of 2D metal oxide and carbon nanosheet is an effective solution to improve the conductivity of the metal oxide [6,7]. 

Recently, we have successfully inserted amine molecules (benzylamine, benzidine, and 3,3′-diaminobenzidine) into the interlayer of 2D-layered TiO_2_ nanosheets through the “wedge reaction”, and subsequently synthesized a layer-by-layer stacked carbon/TiO_2_ nanosheet superlattice composite with an excellent capability as the electrodes for LIB [8,9]. Well-defined interlayered graphene-like carbon nanosheets have been prepared after in situ polymerization and carbonization of amine molecular monomers between 2D-layered metal oxide. However, the carbothermal reaction in the final carbonization process needs a step of sintering the carbon precursor into the stable graphene-like carbon nanosheets, which leads to the reduction of the doped Ni and TiO_2_, resulting in an unclear lithium-ion storage mechanism in the 2D-layered carbon/TiO_2_ superlattice composites.

Recently, as Ti^3+^ have been reported to exhibit a faster electron transfer rate than Ti^4+^, it is attractive to enhance the conductivity of TiO_2_ by the partial reduction of Ti^4+^ [10,11,12]. Meanwhile, it is well known that doping nitrogen atoms with relatively high electronegativity and a small atomic radius into the host carbon is beneficial to enhance the insertion of lithium-ions, and then affects the charge and discharge capability in the process of the electrochemical lithium storage [13]. On the other hand, the introduction of nitrogen as a donor state can improve the conductivity of the carbon materials [14,15,16,17,18,19,20,21]. Moreover, nitrogen doping can be obtained by in situ growth of small nitrogen-containing molecules and carbon sources, or by direct thermal decomposition of nitrogen-containing organic precursors. Peng et al. have reported using melamine and pitch as nitrogen sources and the carbon precursors to synthesize N-doped 2D carbon nanosheets by a molten salt method for LIB, which demonstrates a high capacity of more than 800 mAh g^−1^ at a current density of 0.1 A g^−1^ [22]. Zheng et al. have reported to directly pyrolyze nitrogen-containing zeolite imidazolate skeleton ZIF-8 into N-doped 3D carbon material under N_2_ [23]. Additionally, an amount as high as 17.7 wt% nitrogen-content in the carbon material can significantly improve the cycle stability of the electrode. Therefore, it is of great significance for carbon precursors to be intercalated between 2D-layered metal oxide nanosheets in the confined polymerization systems, in order to increase the conductivity of the composites.

Herein, we demonstrate that defect engineering can be used to improve the electrochemical properties of layered carbon/TiO_2_ superlattice composites. By precise control of the polymerization and carbothermal atmospheres, low valence titanium in metal oxide and nitrogen-doped carbon nanosheets can be obtained in the carbon/TiO_2_ superlattice composite. By optimizing the polymerization and carbonization conditions of the interlaminar p-phenylenediamine (p-PDA) molecules under different atmospheres (N_2_, N_2_/H_2_, and H_2_), p-PDA is successfully converted into a stable N-doped carbon nanosheet between 2D-layered metal oxide after the carbothermal reaction. Moreover, the formation of Ti^3+^ defects is a conductive favored in the lattice-distorted metal oxide layer, which further enhances the electrochemical lithium storage properties. The main framework and interlayer regulation of this kind of metal oxide/carbon composite structure provide experience for the construction of various self-assembled 2D-layered metal oxide/carbon nanosheet superlattice composites.

## 2. Materials and Methods

### 2.1. Material Preparation

Benzylamine/Ni-doped TiO_2_ (benzylamine/NTO) was used as the start material according to our previous report [8,9]. P-phenylenediamine/NTO was prepared by the molecular exchange reaction of p-phenylenediamine (1 g) with benzylamine/NTO (1 g) at 100 °C for 5 h in water (60 mL). The produced P-phenylenediamine/NTO was then subjected to pretreatment at 200 °C for 6 h in the O_2_ atmosphere. The temperature was then raised to 400 °C and treated for 6 h to obtain Poly-p-PDA/NTO. Next, the Poly-p-PDA/NTO was subjected to heat treatment at 500 °C for 3 h at N_2_, N_2_/H_2_ or H_2_ atmosphere, respectively. Finally, three carbon/TiO_2_ composites were obtained after the cleaning of the reduced Ni particles in the structure with 0.1 M HCl acid solution and a large amount of distilling water.

### 2.2. Electrochemical Characterization

The electrode slurry was prepared by mixing 80 wt% active material (carbon/TiO_2_), 10 wt% Vulcan carbon XC-72 (Cabot, Billerica, MA, USA) and 10 wt% polyvinylidene fluoride (HSV900, Kejing, Hefei, Anhui, China)/N-methyl-2-pyrrolidone (NMP, Dodochem, Suzhou, Jiangsu, China, anhydrous, 99.5%). The slurry was coated onto a copper foil and dried at 110 °C in a vacuum oven overnight. Electrochemical tests were carried out with a standard CR2032 button cell. Lithium metal and Celgard 2500 (Dodochem, Suzhou, Jiangsu, China) were used as the counter electrode and the diaphragm, respectively. The mass loading of the active material was about 2 mg cm^−2^. The electrolyte solution was 1.0 M LiPF_6_ in ethylene carbonate/diethylene carbonate (*v*/*v* = 1:1) (water < 10 ppm). The constant current charge/discharge cycle tests were conducted on the Neware battery cycler (Shenzhen Neware, Shenzhen, China). Cyclic voltammetry measurement was conducted on the CHI 760E electrochemical workstation (Shanghai Chenghua, Shanghai, China).

## 3. Results

P-PDA molecules were inserted into layered Ni-doped TiO_2_ nanosheets to prepare p-phenylenediamine/Ni-doped TiO_2_ (p-PDA/NTO) by following a similar method, as reported in our previous work [9]. After exploring the oxidative polymerization methods under different conditions, a regular and strictly layered structure was obtained for the poly-p-phenylenediamine/Ni-doped TiO_2_ (poly-p-PDA/NTO) composite. The polymerized samples were then subjected to carbonization treatments in different atmospheres (N_2_, N_2_/H_2_, and H_2_) and treated with an acid solution to remove the reduced Ni particles in the structures.

**Figure 1:**
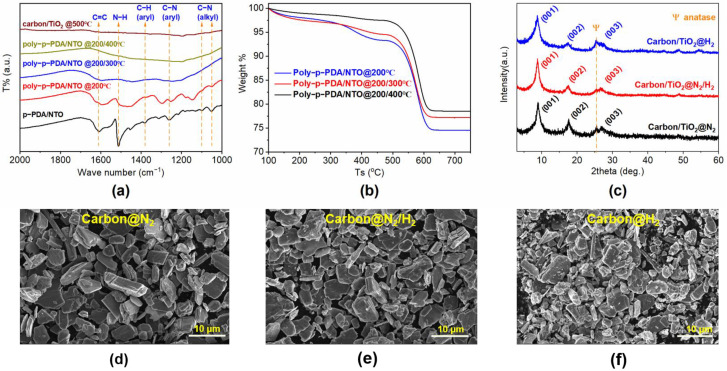
(**a**) FTIR data for the samples before and after the polymerization treatments under different conditions; (**b**) TG curves for the samples of poly-p-PDA/NTO under different treatment temperatures; (**c**) XRD patterns and (**d**–**f**) SEM images of the carbon/TiO_2_ composites after the treatments in different gases.

The degree of polymerization can be judged by the detailed characterization of the functional groups in the organic molecules between metal oxide layers. Figure 1a is the Fourier transform infrared spectra (FTIR) data for the samples before and after the polymerization treatments under different conditions. In detail, the p-PDA/NTO exhibits a C-N stretching vibration peak (alkyl) at 1050 and 1100 cm^−1^, a C-N stretching vibration peak (aryl) at 1260 cm^−1^, and an N-H bending vibration peak of the amino group on the aromatic ring at 1512 cm^−1^ is also displayed. With the increase of the polymerization temperature, the strength of the N-H bond decreases significantly, which indicates that amino groups in phenylenediamine are coupled to each other during the polymerization process. Moreover, full polymerization of p-PDA molecules under O_2_ can reduce the loss of carbon in the subsequent high-temperature carbonization process. However, it is worth noting that self-oxidation will lead to the degradation of organic matter. At a higher processing temperature, poly-p-PDA/NTO is severely self-oxidized, and the degradation of interlayer organic matter causes the layer spacing to decrease, and at the same time the layered TiO_2_ is partially transformed into anatase particles, resulting in the collapse of the layered structure. Therefore, it is particularly important to obtain the p-PDA/NTO samples with a higher polymerization degree and less oxidative degradation as much as possible. The thermogravimetric analysis (TG) curve in Figure 1b shows that the carbon nanosheet in the composite with a lower polymerization temperature has a relatively lower thermal stability, and poly-p-PDA/NTO samples at 200 and 200/300 °C all show an obvious weight loss below 400 °C in O_2_ atmosphere, which is due to the incomplete oxidative polymerization and the evaporation of free amino/aromatic rings between metal oxide layers at the lower temperatures. However, the weight loss for poly-p-PDA/NTO at 200/400 °C is negligible until 600 °C, indicating full oxidation with a two-step polymerization at 200/400 °C. Moreover, the carbon content in poly-p-PDA/NTO at 200/400 °C is similar to the other samples except for the influence of water.

Taking into account the advantages of heteroatom doping and oxygen defects into the electrode material for increasing energy storage sites and conductivity, in this work, the sample of poly-p-PDA/NTO at 200/400 °C are subjected to carbonization treatments under three different gases (N_2_, N_2_/H_2_, H_2_) to carry out defect modification. Three kinds of carbon/TiO_2_ composites can be obtained after the removal of Ni elements through the acid treatment of the sample after carbonization, as different carbonization conditions (N_2_, N_2_/H_2_, H_2_) can calibrate the defective structure of 2D-layered carbon/TiO_2_ superlattice composites, which can further affect their electrochemical lithium storage properties. In the process of carbonization, N_2_ can avoid the loss of heteroatom N in the carbon nanosheet to the greatest extent, which can produce a large number of topological defects and improve the conductivity of the composite as well as the intercalation properties of lithium ions [24]. Although it is not conducive for nitrogen doping in the reduction system, when H_2_ is present in the carbonization atmosphere, Ti^4+^ in layered TiO_2_ can be partially reduced to low-valent titanium dioxide (Ti^x+^, x < 4), and the reduced Ni particles endow a large number of oxygen vacancies in the lattice of metal oxide; both of them can improve the conductivity of the composite material. Figure 1c is the X-ray diffraction (XRD) patterns of the carbon/TiO_2_ composites after the treatments in different gases (N_2_, N_2_/H_2_, H_2_), which are abbreviated as carbon/TiO_2_@N_2_, carbon/TiO_2_@H_2_, and carbon/TiO_2_@ N_2_/H_2_, respectively. The typical diffraction peaks of 001, 002, and 003 in the XRD patterns for each sample represent the regular layered structure, indicating the structure of carbon/TiO_2_ composite is stable during the carbonization process under different treatment atmospheres. As the Ni elements are precipitated during the carbonization treatment under the reducing atmosphere and can be washed away in the subsequent acidification process, lots of oxygen defects are introduced into the lattice of the layered TiO_2_. However, a weak diffraction peak of the anatase phase can be noticed from the XRD patterns as the defects caused by the reduction of Ni atoms induce the rearrangement of TiO_6_ octahedron in the layered TiO_2_, resulting in a small amount of the anatase phase. Figure 1d–f and Figure S1 represent the scanning electron microscope (SEM) images of the layered materials before and after the carbothermal reaction under three different gases. Generally, there is no obvious morphology difference for the three carbon/TiO_2_ superlattice composites.

**Figure 2:**
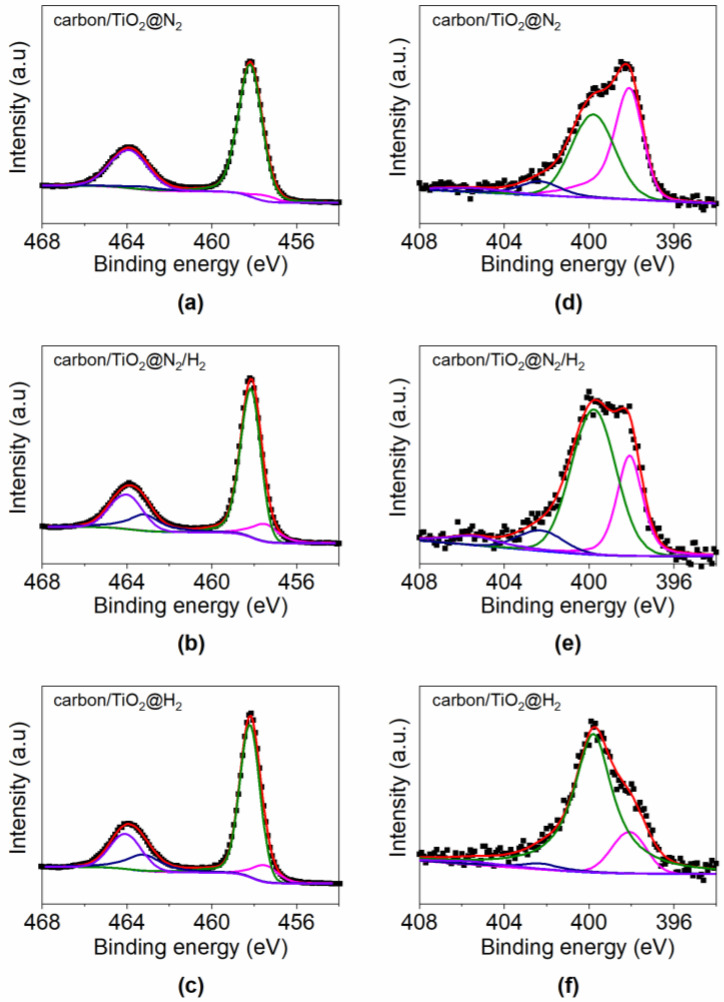
The XPS spectra of (**a**–**c**) Ti 2p and (**d**–**f**) N 1s for the carbon/TiO_2_ composites.

The X-ray photoelectron spectroscopy (XPS) spectra of Ti 2p and N 1s for the carbon/TiO_2_ composites are shown in Figure 2. The band on the Ti 2p spin-orbital of carbon/TiO_2_ composites can be deconvolved into a characteristic peak with a Ti 2p3/2 binding energy center at 458.2 eV and 464.0 eV; this is a typical characteristic of Ti^4+^ [25]. In addition to the shoulder peak at lower binding energies, the Ti of carbon/TiO_2_@ N_2_/H_2_ and carbon/TiO_2_@H_2_ exhibit obvious Ti^3+^ splitting signals at the binding energy positions of 457.5 eV and 463.2 eV, respectively. The XPS spectra of Ti 2p confirm that Ti^4+^ in TiO_2_ have been partially reduced to Ti^x+^ (x < 4) at the presence of H_2_ gas. This increase in the Ti^3+^ concentration can be attributed to the existence of lattice distortion, which allows for more accessible sites for the reduction of Ti^4+^ to Ti^3+^ under H_2_. The metal oxide layer in the 2D carbon/TiO_2_ composite is also rich in oxygen vacancies due to the formation of reduced titanium dioxide [26,27]. Figure 2d–f exhibits the deconvoluted N 1s core level spectra for the carbon/TiO_2_ composites. Four different N-containing functional peaks can be found from the N 1s peaks, termed as the pyridinic N peak at 398.1 eV, pyrrolic/pyridine N peak at 399.8 eV, quaternary N peak at 402.3 eV and oxidized N peak at 405.5 eV. No contribution of amine or amide has been found, indicating a good polymerization and carbonization of the p-PDA precursor molecules. Among four types of N-doped carbon sites, the pyridinic type N has been reported to exhibit a good lithium storage capacity [28,29,30]. Comparing the pyridinic N of the three samples, the amount of pyridinic N in carbon/TiO_2_@H_2_ is the lowest, indicating a nitrogen loss for the carbon/TiO_2_ with the treatment under the H_2_ atmosphere. Notably, the intensity of oxidized N peak in carbon/TiO_2_@N_2_/H_2_ is higher than others, which is in coexistence with the reduced Ti^3+^ in the composite as shown in the fitting result of Ti 2p, indicating a good structure stability of Ti^3+^ in the carbon/TiO_2_@N_2_/H_2_.

**Figure 3:**
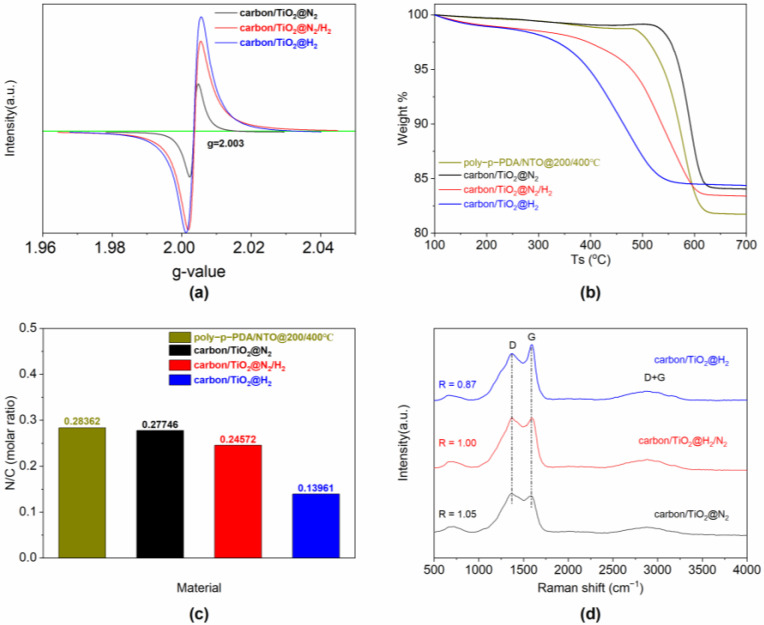
(**a**) EPR spectroscopy for various carbon/TiO_2_ composites; (**b**) TG curves for poly-p-PDA/NTO, carbon/TiO_2_@N_2_, carbon/TiO_2_@N_2_/H_2_, and carbon/TiO_2_@H_2_ in air; (**c**) N/C molar ratio in carbon/TiO_2_ composites measured by elemental analyzer; (**d**) Raman spectra for various carbon/TiO_2_ composites.

Additionally, oxygen defects were also studied by using electron paramagnetic resonance spectroscopy (EPR), and the g value of the oxygen radical derived from the EPR spectroscopy (Figure 3a) is 2.003, a typical characteristic of the single-electron capturing oxygen vacancies [27], which further verifies the generation of rich oxygen vacancies in layered TiO_2_ due to the partial transition of Ti^4+^ to Ti^x+^ (x < 4). The intensity of carbon/TiO_2_@N_2_/H_2_ and carbon/TiO_2_@H_2_ at g = 2.003 is higher than that of carbon/TiO_2_@N_2_, indicating a higher reduction activity for carbon/TiO_2_ after the carbonization treatment at the presence of H_2_. Meanwhile, carbon/TiO_2_@H_2_ demonstrates the highest signal intensity of oxygen radicals among the three composites. Moreover, the heat treatment atmosphere is important for the polymerization and carbonization of anilines to derive N-doped carbon materials. The content of the nitrogen element in the carbon material will decrease as some amino groups in the N-doped carbon layer react to form volatile ingredients (such as NH_3_) in the presence of H_2_ gas, whereas, the number of defects increases in the carbon skeleton of carbon/TiO_2_ composites due to the consumption of nitrogen. TG curves in Figure 3b show the thermal stability of the carbon/TiO_2_ composites. Under the oxidative treatment, the sample of carbon/TiO_2_@H_2_ begins to lose weight at 226.0 °C, and the corresponding temperature of carbon/TiO_2_@N_2_/H_2_ is 312.2 °C, which is 510.8 °C for carbon/TiO_2_@N_2_. In comparison, the carbon content of poly-p-PDA/NTO, carbon/TiO_2_@N_2_, carbon/TiO_2_@N_2_/H_2_, and carbon/TiO_2_@H_2_ are 17.0%, 14.9%, 15.4%, and 14.4%, respectively. Nonetheless, the samples of carbon/TiO_2_@N_2_ and carbon/TiO_2_@N_2_/H_2_ maintain a high N/C ratio during the carbonization process at the presence of N_2_. The values of the N/C molar ratio in carbon/TiO_2_ composites (Figure 3c) measured by the elemental analyzer further confirm the inevitable loss of small molecular monomer and the necessity of N_2_ during the carbonization process. Raman spectra of the composites are also performed and the results are shown in Figure 3d. The Raman peaks for these carbon/TiO_2_ composites are relatively wide, indicating a structural disorder [31]. Generally, the D band at 1372 cm^−1^ and the G band at 1590 cm^−1^ represent the degree of defect and graphitization, respectively. Therefore, the R-value (ID/IG, the ratio of integrated intensity of the D band to the integrated intensity of the G band) of the sample for carbon/TiO_2_@N_2_ and carbon/TiO_2_@N_2_/H_2_ exhibit a similar crystalline structure, as shown in Figure 3d.

To compare the conductivity difference of the carbon/TiO_2_ composites after the treatments in different gases, a blocked cell was used to evaluate the total conductivity of the samples; the corresponding results are shown in Figure S2. The conductivity of carbon/TiO_2_@N_2_/H_2_ is the highest with a value of 1.51 mS cm^−1^, while the conductivity of carbon/TiO_2_@H_2_ and carbon/TiO_2_@N_2_ is 0.47 mS cm^−1^ and 0.029 mS cm^−1^, respectively, which are one or two orders of magnitude lower than that of carbon/TiO_2_@N_2_/H_2_. It is known that Ti^x+^ (x < 4) with a low chemical valence can greatly improve the electron transport rate. Moreover, due to the generation of reduced titanium, part of O^2−^ in TiO_2_ lattice is converted into O_2_ and removed, making the 2D metal oxide layer rich in a large number of oxygen vacancies, which further improves the electronic conductivity. Moreover, the protection of N_2_ to N-doped carbon layer in the carbon/TiO_2_ composite also results in the highest conductivity for carbon@N_2_/H_2_. However, there is no similar comprehensive structure to improve conductivity for carbon/TiO_2_@N_2_ and carbon/TiO_2_@ H_2_. The absence of reduced titanium in carbon/TiO_2_@N_2_ makes its conductivity the lowest one among the three carbon/TiO_2_ composites. Therefore, the highest conductivity is finally achieved for carbon/TiO_2_@N_2_/H_2_ among the three composites.

**Figure 4:**
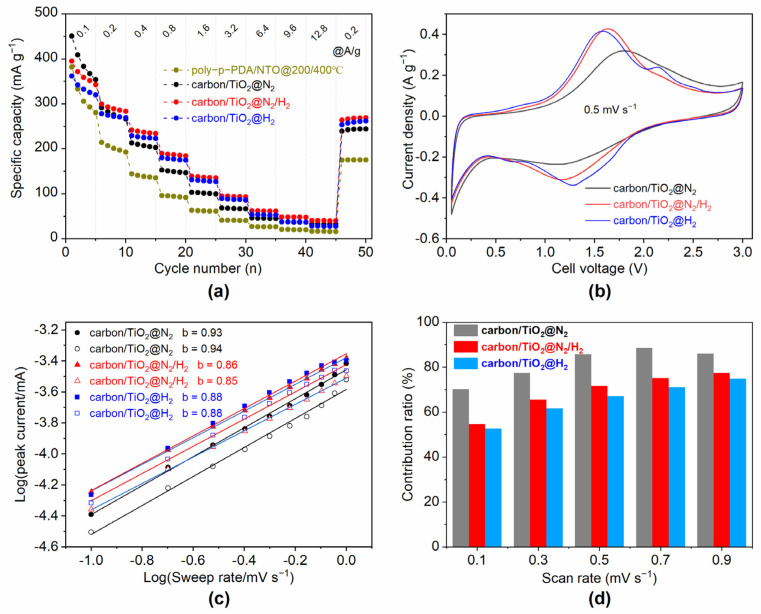
(**a**) Rate capabilities for poly-p-PDA/NTO, carbon/TiO_2_@N_2_, carbon/TiO_2_@N_2_/H_2_, and carbon/TiO_2_@H_2_; (**b**) the cyclic voltammogram curves for carbon/TiO_2_@N_2_, carbon/TiO_2_@N_2_/H_2_, and carbon/TiO_2_@H_2_; (**c**) relationship between the peak currents and scan rates in logarithmic format; (**d**) contribution ratio of the capacitive and diffusion-controlled charge storage at different scan rates for carbon/TiO_2_ composites.

Nevertheless, as reported in previous studies [32,33], the high-speed intercalation/deintercalation ability of Li^+^ in regular layered structures is related to sufficient active sites in the electrode materials. It is well known that the N-doped carbon layer can stabilize the interface between the electrode and the electrolyte during the charge/discharge cycling [33]. The conductivity improved by reductive titanium dioxide is also beneficial to the charge and discharge capacity at different rates. To check the lithium storage and rate capability of the 2D-layered carbon/TiO_2_ composites, standard CR2032 coin cells were assembled with metallic Li as the counter electrode. Figure 4a and Figure S3 show the rate capabilities and the corresponding charge/discharge curves of carbon/TiO_2_ composites at the current densities ranging from 0.1 A g^−1^ to 12.8 A g^−1^ within an electrochemical voltage window between 0.05 and 3 V. As a reference, the electrochemical performances for the sample of poly-p-PDA/NTO composite were also checked under the same conditions. The comparative performance of the precursor-layered titanic acid and graphite is presented in our previous work [9]. The low Coulomb efficiency (carbon/TiO_2_@N_2_ = 50.6%, carbon/TiO_2_@N_2_/H_2_ = 51.2%, and carbon/TiO_2_@H_2_ = 49.0%) at the first charge-discharge cycle for all the samples could be attributed to the irreversible formation of a solid electrolyte interfacial film (SEI) on the surface of the lithium anode due to the decomposition of electrolyte. This process at the electrode/electrolyte interface usually reacts irreversibly on the first several cycles until forming a stable SEI layer, which is a necessary component for lithium-ion batteries. The role of the SEI layer involves the prevention of further electrolyte decomposition to maintain cycling ability. During the second cycle, the discharge capacity decreases to 450.7, 361.6, and 395.6 mAh g^−1^ with a corresponding charge capacity of 489.8, 361.5, and 409.0 mAh g^−1^ for carbon/TiO_2_@N_2_, carbon/TiO_2_@H_2_, and carbon/TiO_2_@N_2_/H_2_, respectively, leading to a high coulombic efficiency. This high coulombic efficiency after the second cycles indicates the good reversibility of lithiation/delithiation processes in the composites. The specific capacities for all carbon/TiO_2_ composites are higher than that of uncarbonized poly-p-PDA/NTO composite at various current densities, as the amount of electrochemically active sites in the carbon/TiO_2_ materials increase after the removal of Ni. Moreover, the regularization and defects of the carbon skeleton, the rich oxygen vacancies in the TiO_2_ layer, and the enhanced conductivity for carbon/TiO_2_ composites are all benefited for the outstanding electrochemical activity. Electrochemical impedance spectroscopy data (Figure S4) for carbon/TiO_2_@N_2_, carbon/TiO_2_@N_2_/H_2_, and carbon/TiO_2_@H_2_ at the high frequency region demonstrates a slightly difference in value dominated by charge transfer for the three materials. However, it can still be demonstrated that the formation products of Ti^3+^ and oxygen vacancies have greater conductivity when H_2_ is present in the carbonization. The specific capacity drops moderately with the increase of current density for all the electrode materials. Carbon/TiO_2_@N_2_/H_2_ exhibits the best rate and capability performance among all the carbon/TiO_2_ electrodes. The discharge capacity for carbon/TiO_2_@N_2_/H_2_ is 396.0, 300.0, 241.7, 189.8, 138.8, 94.7, 61.8, and 48.0 mAh g^−1^ at the current of 0.1, 0.2, 0.4, 0.8, 1.6, 3.2, 6.4, and 12.8 A g^−1^, respectively. Moreover, after turning back the current density from 12.8 A g^−1^ to 0.2 A g^−1^, the specific capacity of carbon/TiO_2_@N_2_/H_2_ rapidly reverts to 270.0 mAh g^−1^, indicating a good stability. The excellent electrochemical performance for carbon/TiO_2_@N_2_/H_2_ can be explained by the existence of sufficient defect sites on the N-doped carbon/TiO_2_ skeleton and the high conductivity of the layered material [34,35]; nitrogen doping in the 2D carbon layer leads to the structural distortion of the ideal graphene structure, while pyridine N formed at edges and defects can promote the vertical diffusion of lithium ions [36,37], thus leading to a high specific capacity. Moreover, the charge distribution on the surface of TiO_2_ treated by H_2_ is more uniform due to the reduced titanium and a large number of oxygen vacancies [38]. The unique nitrogen doping in the carbon and reduced Ti^3+^ in the metal oxide contribute to the overall high electronic conductivity of the material, which greatly improves the Li deintercalation/intercalation kinetics under different current densities. Notably, without the high conductivity of Ti^x+^ (x < 4), carbon/TiO_2_@N_2_ demonstrates the lowest specific capacity and rate stability at all currents.

To distinguish the reaction mechanisms during the electrochemical process, electrochemical cyclic voltammetry (CV) tests were carried out in the voltage range of 0.05 V–3 V to evaluate the lithium-ion diffusion kinetics in the carbon/TiO_2_ composites. Figure 4b shows cyclic voltammograms for the three carbon/TiO_2_ composites at a scan rate of 0.5 mV s^−1^. The redox peaks appearing at 1.78/1.16 V, 1.62/1.20 V, and 1.57/1.28 V correspond to the intercalation/deintercalation of Li^+^ in the TiO_2_ lattice of carbon/TiO_2_@N_2_, carbon/TiO_2_@N_2_/H_2_, and carbon/TiO_2_@H_2_, respectively. Moreover, the peak intensity and integral area of carbon/TiO_2_@N_2_/H_2_ and carbon/TiO_2_@H_2_ at the peak current are higher than the value of carbon/TiO_2_@N_2_, which is mainly due to the increase of conductivity by the reduced Ti^x+^ (x < 4) in the composites [39]. Meanwhile, the potential difference of the cathode/positive peaks for carbon/TiO_2_@N_2_ is larger than that of carbon/TiO_2_@N_2_/H_2_ and carbon/TiO_2_@H_2_, which is due to the higher polarization phenomenon resulted by the worse conductivity of carbon/TiO_2_@N_2_, as shown in Figure S2. The phenomenon is consistent with the result of the rate performance. It is worth noting that a unique anode peak appears at the CV curve of carbon/TiO_2_@H_2_ at around 2.12 V, which is possibly due to the existence of the higher content of anatase in carbon/TiO_2_@H_2_. However, the existence of anatase particles is controlled by diffusion during energy storage, rather than the capacitive type (Figure S5), thus resulting in a slower rate capability for carbon/TiO_2_@H_2_ versus carbon/TiO_2_@N_2_/H_2_.

The charge storage contribution of diffusion/capacitive can usually be judged according to the formula *i* = a*v*^b^, where *i* (mA) represents the peak current, *v* (mV s^−1^) represents the scan rate, and a and b represent two adjustable parameters according to the previous report [40,41,42]. When b is 0.5 or 1.0, this represents that the electrochemical lithium storage is controlled by diffusion and capacitance, respectively. Figure 4c is a plot of the linear relationship between the cathode/anode peak currents at different scan rates for the three carbon/TiO_2_ composites. It can be observed from the data that the b values of the cathode/anode peaks for carbon/TiO_2_@N_2_, carbon/TiO_2_@N_2_/H_2_, and carbon/TiO_2_@H_2_ are 0.94/0.93, 0.85/0.86, and 0.88/0.88, respectively. Therefore, the charge storage mode in carbon/TiO_2_@N_2_, carbon/TiO_2_@N_2_/H_2_, and carbon/TiO_2_@H_2_ is mainly dominated by capacitive contribution. To further quantitatively analyze the contribution of capacitive to the total capacity, the formulas *i*(V) = k_1_*v* + k_2_*v*^1/2^ and *i*(V)/*v*^1/2^ = k_1_*v*^1/2^ + k_2_ are used for data fitting to determine the values of K_1_ and K_2_, and then the contribution ratio of capacitance is quantitatively analyzed [40]. The contribution rate of capacitive and diffusion controlled charge storage for carbon/TiO_2_@N_2_, carbon/TiO_2_@N_2_/H_2_, and carbon/TiO_2_@H_2_ at different scan rates are shown in Figure 4d and Figure S6, the peak values of the capacitive contribution ratio for carbon/TiO_2_@N_2_, carbon/TiO_2_@N_2_/H_2_, and carbon/TiO_2_@H_2_ are 88.6%, 78.6%, and 78.4%, respectively. With the increase of scan rate, the proportion of the capacitive contribution for all the carbon/TiO_2_ composites slowly increases, which demonstrates a faster power output for the three carbon/TiO_2_ composites in Li^+^ transmission.

**Figure 5:**
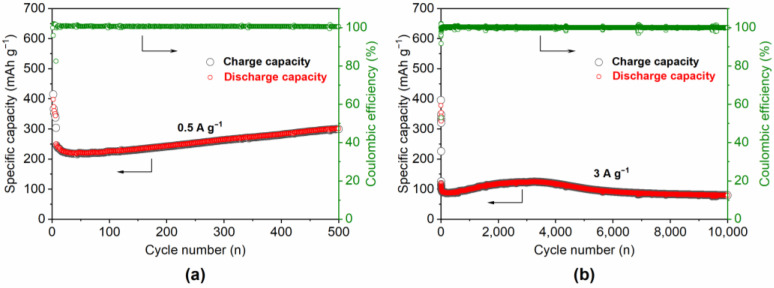
The cycling performance of the coin cell with carbon/TiO_2_@N_2_/H_2_ composite at a current density of (**a**) 0.5 and (**b**) 3 A g^−1^.

The good rate performance of the carbon/TiO_2_@N_2_/H_2_ composite was further demonstrated by cycling the coin cells in the voltage range of 0.05–3 V. The cells were firstly activated for five cycles at a current density of 0.1 A g^−1^ before switching to 0.5 A g^−1^ for the cycle stability tests (Figure 5a). A quick drop of the capacity can be found for all composites, which is attributed to the formation of SEI and the gradual activation of electrochemically active sites in the structure with the cycling [22,43,44]. The discharge specific capacities at the six cycles are 343.7 mAh g^−1^ for carbon/TiO_2_@N_2_/H_2_. However, the discharge capacity continuously drops within the first 30 cycles and then slightly increases with the following 500 cycles (Figure 5a). For carbon/TiO_2_@N_2_/H_2_, the discharge capacity was finally stabilized at 301.6 mAh g^−1^. Moreover, high coulomb efficiency of ≈100% still maintains for the composite, indicating an excellent cycling stability. The stable and stacked 2D-layered carbon/TiO_2_ structure is beneficial in cycle stability. Additionally, Figure 5b is the cycle stability of carbon/TiO_2_@N_2_/H_2_ at a high current density of 3.0 A g^−1^. In the beginning 35 cycles, the capacity slightly decreases to 94.1 mAh g^−1^, and then slowly increases to 118.0 mAh g^−1^ in the following 2000 cycles. Afterward, the charge/discharge specific capacity begins to decay slowly as the number of cycles increases. However, a high discharge capacity of 80.0 mAh g^−1^ is still maintained after 10,000 cycles, which corresponds to the capacity retention rate of 85%. The rich O vacancies in the 2D-layered carbon/TiO_2_@N_2_/H_2_ and the defects in the 2D N-doped carbon nanosheet provide sufficient interface energy storage spaces and electrochemically active sites. The “sandwich” type layer-by-layer stacked nano-structure also greatly promotes the insertion/extraction of lithium ions in the confined space between the interlayer. Therefore, the structured regulation in different carbonization atmospheres is very beneficial to obtain excellent cycle stability. There is a wide application space in LIB after doping control and interlayer modification on the substrate of 2D metal oxide/carbon nanosheets with a regular structure. However, the loss of the small molecules during the high-temperature carbonization reaction inevitably leads to the inevitable decline of the capacity and conductivity. It is still a great challenge to realize 2D-layered metal oxide/carbon nanosheet superlattice composites with high energy density and high-power density.

## 4. Conclusions

In summary, 2D-layered carbon/TiO_2_ composite with a high conductivity has been synthesized by tuning the defects of low valence titanium in the metal oxide and nitrogen species in the carbon nanosheet under a processing atmosphere of N_2_/H_2_ gas. The resultant composite exhibits an excellent rate capability and good cycle stability. The superior electrochemical performance can be ascribed to the existence of reduced titanium and the high amount of N-doped carbon nanosheet in the 2D-layered carbon/TiO_2_, which not only increases the electronic conductivity of the material but also provide sufficient electrochemical active sites. Moreover, the 2D-layered structure provides enough lithium-ion transport channels and interface capacitance in the process of quick lithium-ion transmission. Defect manipulation in a confined space provides a new direction for the modification of a wide range of 2D materials. The prepared 2D carbon/TiO_2_ composite can provide a high capacity of 396.0 mAh g^−1^ at a current density of 0.1 A g^−1^. Moreover, a high specific capacity of 80.0 mAh g^−1^ with the capacity retention rate of 85% remains after 10,000 charging/discharging cycles at 3.0 A g^−1^ as well as the Coulomb efficiency close to 100%. The nanosheet composite with orderly arrangement is expected to be one of the promising anodes for the next generation lithium-ion batteries. It is believed that the intriguing layered superlattice structure can be further extended to the preparation of many other kinds of N-doped carbon nanosheet/metal oxide nanosheet superlattice composites for wide applications, including energy storage for alkaline-ion batteries.

## Data Availability

The data presented in this study are available on request from the corresponding author.

## Supplementary Materials

The following supporting information can be downloaded at: https://www.mdpi.com/article/10.3390/ma15051625/s1, chemicals; materials characterization; electrical resistances measurements; Figure S1: SEM image of Poly-p-PDA/NTO@200/400°C; Figure S2: (a) The optical picture of the two-electrodes blocked cell. (b) The electronic conductivity of carbon/TiO2@N2, carbon/TiO2@N2/H2, and carbon/TiO2@H2; Figure S3: Charge/discharge curves of carbon/TiO2@N2, carbon/TiO2@N2/H2, and carbon/TiO2@H2 under the current densities of 0.1, 0.2, 0.4, 0.8, 1.6, 3.2, 6.4, 9.6, and 12.8 A g−1; Figure S4: The impedance spectra of carbon/TiO2@N2, carbon/TiO2@N2/H2, and carbon/TiO2@H2; Figure S5: b-values and the relationship between the anode peak current and the scan rate of carbon/TiO2@H2 in a logarithmic format at about 2.1 V; Figure S6: Contribution ratio of the capacitive and diffusion-controlled charge storage at 0.1, 0.3, 0.5, 0.7, and 0.9 mv s−1 for carbon/TiO_2_@N_2_, carbon/TiO_2_@N_2_/H_2_, and carbon/TiO_2_@H_2_.
