# Supplementary material for: Tuning the Defects of Two-Dimensional Layered Carbon/TiO2 Superlattice Composite for a Fast Lithium-Ion Storage
Source: Materials (Basel). 2022 Feb 22;15(5):1625. doi: 10.3390/ma15051625 (PMC8911284; doi:10.3390/ma15051625)
Supplement: Supplementary file 1 [file materials-15-01625-s001.zip › materials-1589469-supplementary.pdf]

materials

Article

Tuning the Defects of Two-Dimensional Layered Carbon/TiO_2 Superlattice Composite for a Fast Lithium-Ion Storage

Bingheng Liu [1,2,3], Bo Gu [2,3], Jingxian Wang [2,3], Anchang Li [2,3], Ming Zhang, [2,3,*] and Zhongrong Shen [1,2,3,*]

Chemicals

Titanium oxide (TiO2, anatase, 99.8%, Aladdin), Nickel oxide (NiO, 99.0%, Aladdin), P-phenylenediamine (C6H8N2，99.5%，Sinopharm Chemical Reagent Co., Ltd.), Potassium carbonate (K2CO3, 99.0%) and benzylamine (C7H9N, 98.5%, Sinopharm Chemical Reagent Co., Ltd.) were used directly without any purification after purchase. TiO2 and K2CO3 were mixed and dried at 120 °C for 24 hours before the experiment.

Materials Characterization

The morphological and texture of the prepared samples were analyzed with X-ray diffraction (XRD, Rigaku RINT 2500, Cu-Kα irradiation, λ=1.5406 Å), Fourier Transform Infrared Spectrometer (FTIR, Thermo Nicolet is50, USA), Scanning Electron Microscopy (SEM, Zeiss Sigma-500), Element analyzer (Vario El Cube), Raman spectra (Labram HR Evolution spectrometer, 532 nm laser source), Electron paramagnetic resonance (EPR, ELEXSYS E500 electron spin resonance spectrometer) and X-ray photoelectron spectroscopy (XPS, Axis Ultra DLD, Kratos Analytical). The thermogravimetric curves were recorded on a Mettler Toledo analyzer from 30 to 800 °C with a heating rate of 5 °C per minute.

Electrical Resistances Measurements

The electrical resistances were determined following the literature by using a blocked cell.1 In detail, 0.2 g carbon/TiO2 was pressed into a pellet under a pressure of 15 Mpa, and then placed between two polished copper electrodes to measure the cyclic voltammetry curve. The electronic conductivity is calculated by the formula σ=L/RS. Where L is the thickness of the pellet, R is the resistance value, and S is the cr

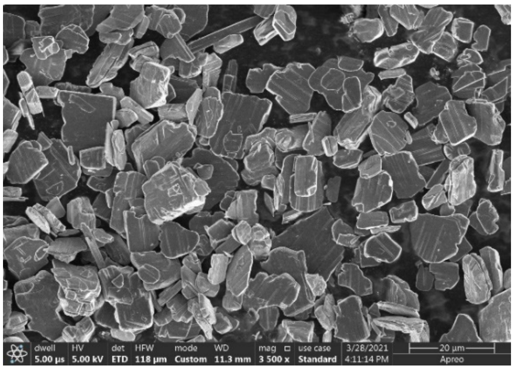

Figure S1. SEM image of Poly-p-PDA/NTO@200/400℃.

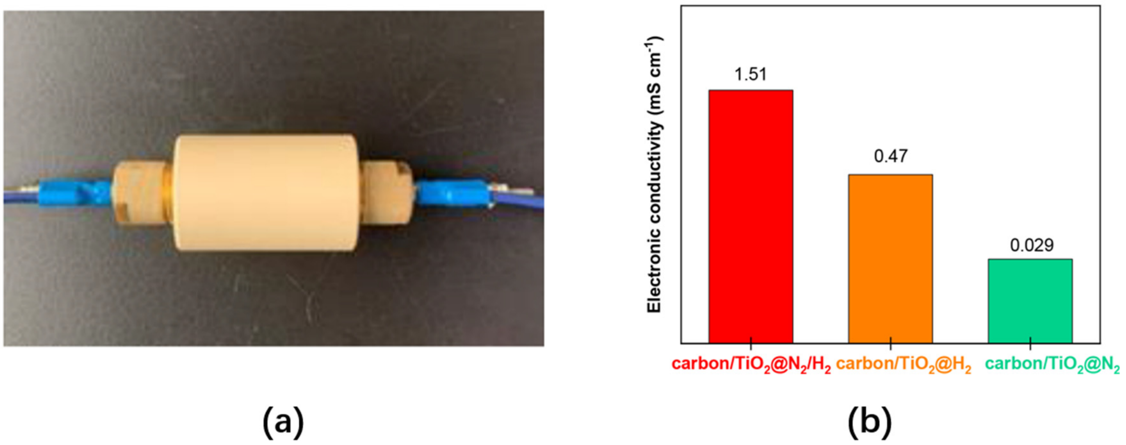

Figure S2. (**a**) The optical picture of two-electrodes blocked cell. (**b**) The electronic conductivity of carbon/TiO_2@N_2, carbon/TiO_2@N_2/H_2 and carbon/TiO_2@H_2.

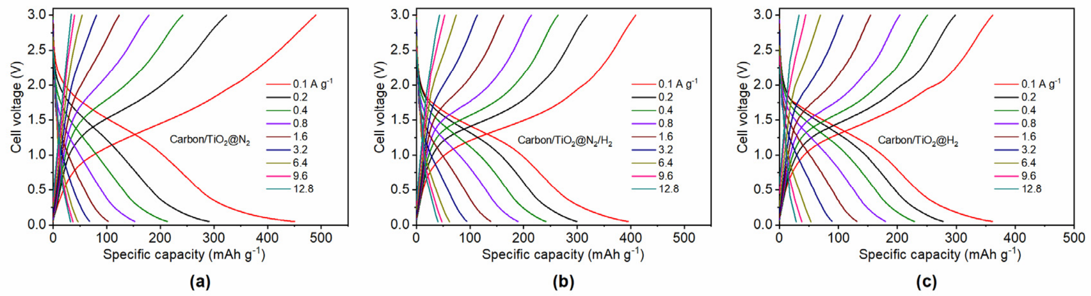

Figure S3. Charge/discharge curves of carbon/TiO_2@N_2, carbon/TiO_2@N_2/H_2 and carbon/TiO_2@H_2 under the current densities.

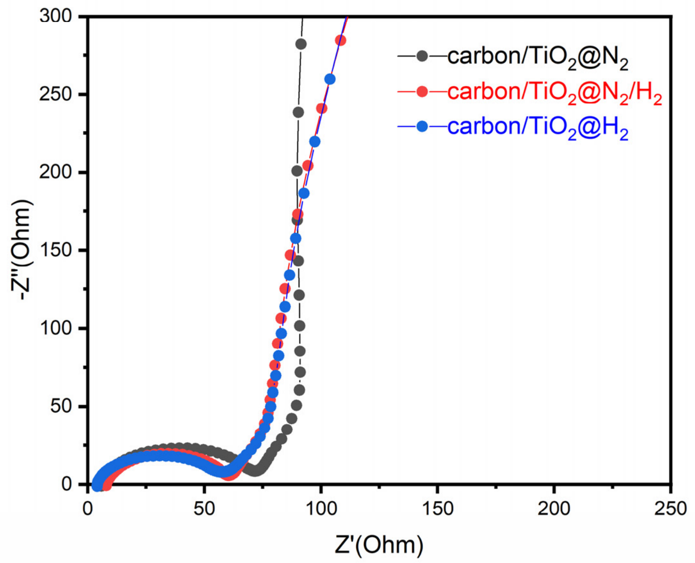

Figure S4. The impedance spectra of carbon/TiO_2@N_2, carbon/TiO_2@N_2/H_2 and carbon/TiO_2@H_2.

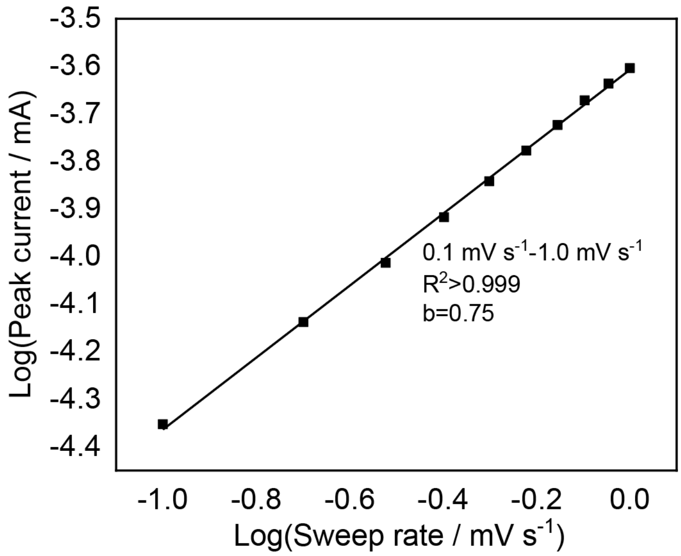

Figure S5. b-values and the relationship between the anode peak current and the scan rate of carbon/TiO_2@H_2 in logarithmic format at about 2.1 V.

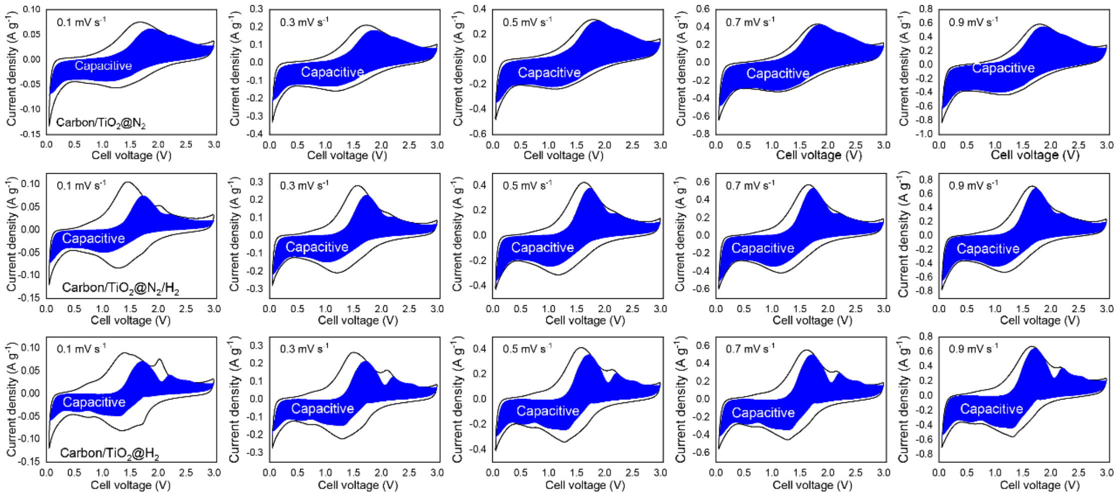

Figure S6. Contribution ratio of the capacitive and diffusion-controlled charge storage at 0.1, 0.3, 0.5, 0.7, 0.9 mv s^{-1} for carbon/TiO_2@N_2, carbon/TiO_2@N_2/H_2 and carbon/TiO_2@H_2.
